# Ultrastructural Comparison of the Nasal Epithelia of Healthy and Naturally Affected Rabbits with *Pasteurella multocida* A

**DOI:** 10.1155/2013/321390

**Published:** 2013-03-14

**Authors:** Paula Esquinas, Lucía Botero, María del Pilar Patiño, Carolina Gallego, Carlos Iregui

**Affiliations:** ^1^Departamento de Patología, Hospital Universitario Fundación Santafé de Bogotá, Bogotá, Colombia; ^2^Laboratorio de Patología Veterinaria, Facultad de Medicina Veterinaria, Universidad Nacional de Colombia. Bogotá, Colombia; ^3^Departamento de Patología Veterinaria, Facultad de Ciencias Pecuarias, Universidad de Ciencias Aplicadas y Ambientales, Bogotá, Colombia

## Abstract

An ultrastructural comparison between the nasal cavities of healthy rabbits and those suffering from two forms of spontaneous infection with *Pasteurella multocida* was undertaken. Twelve commercially produced rabbits of different ages and respiratory health status were divided into four groups: healthy from 0 to 21 days (G1, *n* = 2); healthy from 23 to 49 days (G2, *n* = 2); healthy from 51 to 69 days (G3, *n* = 2); diseased rabbits with septicemia and the rhinitic form of *P. multocida* infection (G4, *n* = 3). The main ultrastructural changes observed were a widening of the interepithelial spaces, increased activity and number of goblet cells, the formation of two types of vacuoles in epithelial cells, the degranulation and migration of heterophils between the epithelial cells, and the association of this migration with some of the other changes. No bacteria were observed adhering to the epithelium, and very few were observed free in the mucus. Scant inter-epithelial spaces were found in healthy rabbits, but they were not as large and numerous as those found in diseased animals. We discuss the origin and meaning of these changes but, we focus on the significance of the inter-epithelial spaces and goblet cells for the defense of the upper respiratory airways against the bacterium and its lipopolysaccharide.

## 1. Introduction

*P. multocida* is commonly associated with different entities of rabbits [1–7]. Clinical signs in rabbits may include rhinitis (snuffles) with purulent nasal discharge, septicemia, pneumonia, otitis media, pyometra, meningitis, and localized abscesses [7–9]. 

The nasal cavity is considered the natural habitat of *P. multocida* in different species, including the rabbit [10]; however, neither the normal ultrastructure of this region nor the pathologic changes during natural infection with *P. multocida* have been described in rabbits. 

Al-Haddawi et al. [11–13] described the gross, histological, and ultrastructural lesions in this anatomical region of rabbits subjected to different experimental infection protocols with *P. multocida* A3,* P. multocida* A3 plus hydrocortisone, and *P. multocida* serotype D1. The experimental animals were killed 14 and 21 days after inoculation. In general, the gross findings in all three studies were congestion of the nasal mucosa, hemorrhages, and/or mucopurulent exudates. Histologically, these authors observed mild to chronic catarrhal and suppurative rhinitis, with few rabbits displaying ulceration of the mucosal surface. Ultrastructurally, they found severely degenerated epithelial cells that were swollen and had vacuolated cytoplasm; these changes involved the mitochondria and rough endoplasmic reticulum, and loss of cilia was also frequently seen. Necrotic cells had both loss of cilia and breakdown of the cell membrane. Polymorphonuclear heterophils and macrophages infiltrated between the degenerated epithelial cells and the subepithelial layer. Finally, goblet cell hyperplasia and hypertrophy were documented. 

On the other hand, histological or ultrastructural studies documenting lesions in the respiratory epithelium of the nasal cavities of rabbits affected with a natural respiratory disease are not frequent. Histologically, we reported various changes in this region in rabbits infected with *P. multocida *[14] including vacuolation of the cytoplasm of epithelial cells and the presence of interepithelial spaces in both healthy rabbits and those infected with *P. multocida, *though these changes were more severe in the latter. Other findings in diseased rabbits were goblet cell hyperplasia and infiltration of heterophils between epithelial cells, the latter of which was associated with damaged epithelial cells. To our knowledge, ultrastructural descriptions of the nasal cavities of healthy rabbits or rabbits with respiratory diseases have not been reported. 

The main purpose of this study was to describe and compare the transmission electron microscopy (TEM) findings of the respiratory epithelium and the propria of the nasal septum from healthy rabbits of different ages under commercial production conditions with those of rabbits affected with the rhinitic and septicemic forms of *P. multocida *type A. 

## 2. Materials and Methods

### 2.1. Bacteriology

After the animals were euthanized, nasal and tracheal swabs as well as lung samples were collected for microbiology. The isolates were cultured in brain-heart agar (BHI) at 37°C for 24 h. Isolates were identified as *P. multocida *type A based on their cultural and biochemical characteristics [15]. For the PCR assay, the primer sequence used for the Gen hyaD-hyaC was 5′ TGC CAA AAT CGC AGT CAG 3′R 5′ TTG CCA TCA TTG TCA GTG 3′ [16].

### 2.2. Animals

Twelve New Zealand White rabbits of different ages from a commercial rabbitry located at the Savannah of Bogotá at 2600 m.a.s., Colombia, were divided into 4 groups: 2 healthy rabbits between 0 and 21 days of age (group 1), 2 healthy rabbits between 23 and 49 days (group 2), 2 healthy rabbits between 51 and 69 days of age (group 3), and 6 acute diseased rabbits, 3 with septicemia (clinically showing cyanosis of the ears and the ocular mucosa, polypnea, dyspnea and orthopnea, and a dilated abdomen) and 3 with rhinitis (group 4). The animals were anesthetized i.m. with 35 mg/kg of ketamine and 5 mg/kg of xylazine then instilled with 2.5% glutaraldehyde (SPI Supplies, West Chester, PA, USA) in Millonig's buffer (4°C) via the nasal cavity. The animals were euthanized with an overdose of anesthesia. The nasal cavity was cross-sectioned at the level of the first molar, and the nasal septum was taken. The samples were divided into two parts: one was immersed in buffered formaldehyde (3.7%) for paraffin embedding and the other in glutaraldehyde (2.5%, 4°C) for TEM. The experimental procedures were approved by the Veterinary School Bioethics Committee of the National University of Colombia. 

### 2.3. Processing and Evaluation of the Samples

Tissues were processed by routine paraffin wax embedding and sectioned at 5 *μ*m. Sections were stained with hematoxylin and eosin (H&E). 

The tissues for TEM were postfixed in osmium tetroxide (1%) and embedded in epoxy resins. Five semithin sections per animal were obtained and stained with toluidine blue. The normal and the pathological morphologies were described, and a 2 mm^2^ area of tissue that included the epithelium, propria, and glandular epithelium was selected for ultrastructural evaluation. Ultrafine sections were made, contrasted with uranyl acetate and lead citrate and evaluated in a Phillips 110 TEM.

## 3. Results

### 3.1. Microbiology

Bacteria could not be isolated from the rabbits in groups 1 and 2, but *P. multocida *was isolated from the nasal cavities and lungs of the animals in groups 3 and 4. The bacteria were identified by PCR as *P. multocida* A. 

### 3.2. Histopathological and Ultrastructural Findings (See Table 1)

#### 3.2.1. Groups 1 (1–21 Days, *n* = 2) and 2 (23–49 Days, *n* = 2)

**Figure 1:**
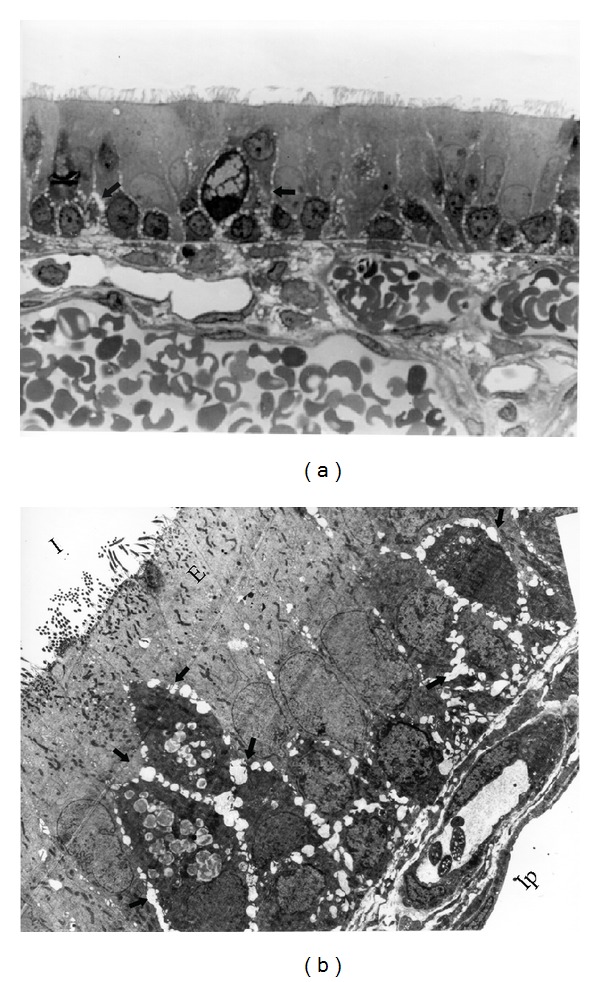
Respiratory nasal epithelium from a rabbit of group 2. (a) Intercellular spaces (arrows) that extent from the basement membrane to approximately 2/3 of the height of the epithelial cells, semi-thin section. 1000x. (b) Electron micrograph of (a): The intercellular spaces (arrows) are more abundant around basal cells. 2500x.

Animals from these groups showed similar findings and will therefore be described together. In the H&E and semithin sections, the rabbits (*n* = 4) did not show major lesions or changes. The most important change was the appearance of intercellular spaces between cells of the respiratory epithelium. These spaces were represented by small dilatations between the cells, without separation at the apical end and were more common on the basolateral side of the cells (Figure 1(a)). Moderate cytoplasmic vacuolation was observed in one animal in group 1. In rabbits of both groups, the epithelial cells of the nasal cavity showed the distribution and ratio that was expected from the other animal species used as references but differed from those of the diseased rabbits. No detritus or exudates were found in the lumen of the cavity. By TEM, the epithelial cells showed the expected morphology, and the presence of interepithelial spaces was confirmed, particularly between the basal cells (Figure 1(b)). Single lymphocytes and heterophils were observed migrating between the epithelial cells, but no changes in the epithelial cells were associated with such a migration. 

#### 3.2.2. Group 3 (51–69 Days) (*n* = 2)

**Figure 2:**
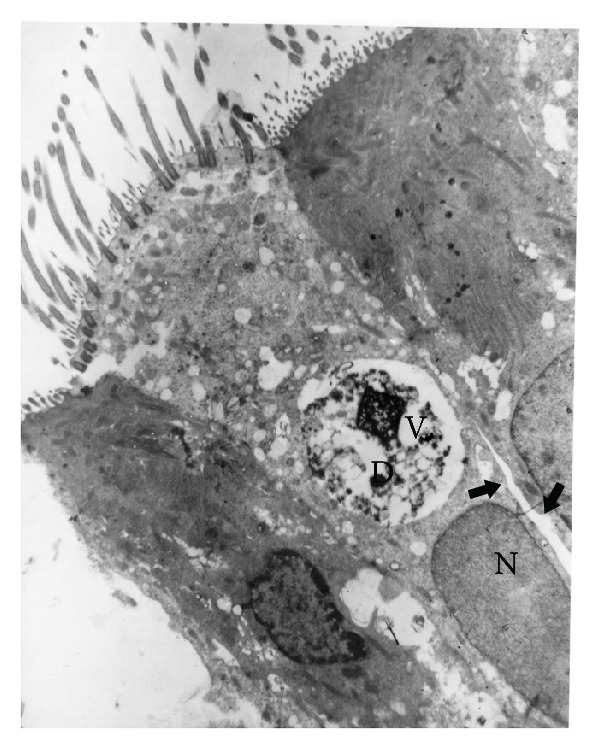
Electron micrograph of respiratory epithelium from a rabbit of group 3. Ciliated cell with electrodense material (D) within a cytoplasmic vacuole, also visible is a severe vacuolation of the cytoplasm. N: nucleus of a ciliated cell. Arrows indicate the interepithelial space. 15500x.

Despite being clinically healthy animals (rabbits at most risk of developing the disease), they showed different types of lesions in varying degrees. Interepithelial spaces were more obvious in these animals and were not limited to the basal region of the cells, with some cells completely separated, detached, and desquamated. Moderate to severe vacuolation of the epithelial cells was also present in both animals. Vacuoles of different sizes were observed, some of which filled the whole cytoplasm. Ultrastructurally, in one rabbit, the epithelium was lower, with apparent loss of cilia and excessive cytoplasmic vacuolation. The vacuoles were of different sizes and contained a membranous or granular material. In the second animal, the ciliated cells showed large vacuoles with granular content, which was heavily electrodense and resembled a typical phagosome in a phagocytic cell (Figure 2). In some places, isolated dead cells (possibly apoptotic) were observed, which were not necessarily associated with inflammatory cells. In others, the epithelial cells had been completely separated, with a loss of tight junctions and the desquamation of some cells. A moderate number of migrating heterophils were associated with these changes in some places.

#### 3.2.3. Group 4, Septicemic Animals (*n* = 3)

**Figure 3:**
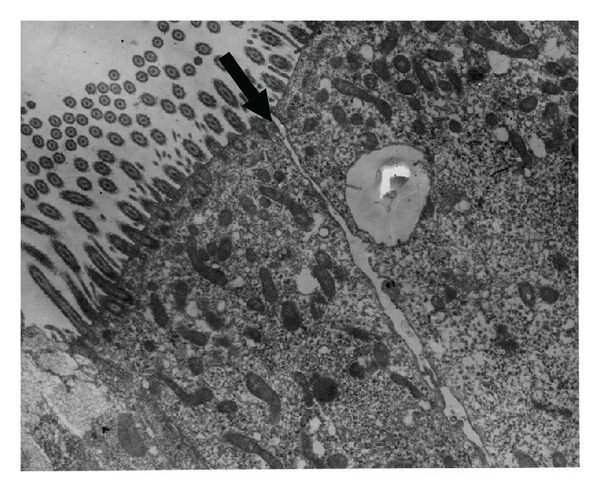
Electron micrograph of nasal epithelium from a rabbit of group 4. Apical opening between two ciliated cells (arrow). The cytoplasm is moderately vacuolated. 10000x.

**Figure 4:**
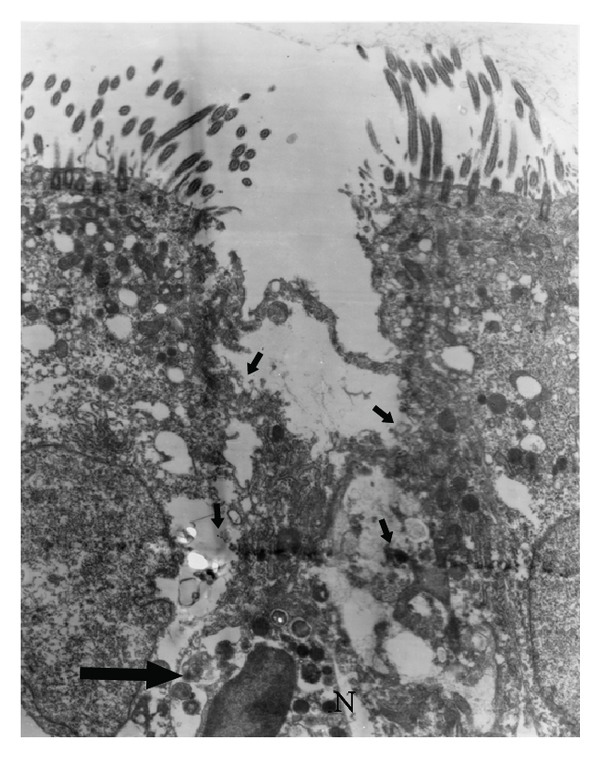
Electron micrograph of nasal epithelium from a rabbit of group 4. A PMN migrating through an intercellular space and separating the apical border of two ciliated cells (arrows). The PMN cytoplasm is less electrodense and with lesser number of granules (large arrow). 5200x.

**Figure 5:**
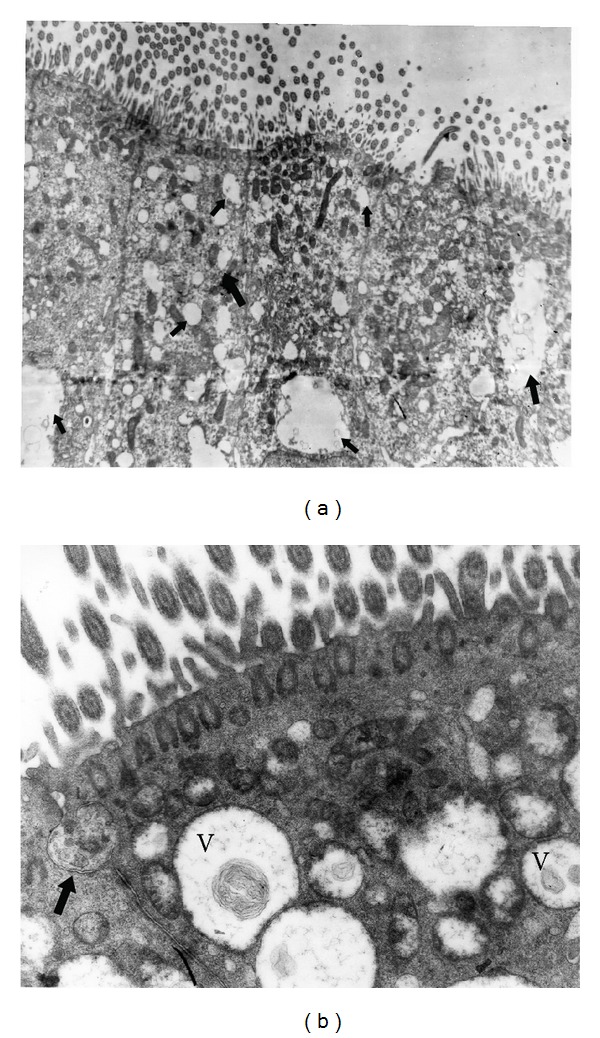
Electron micrograph of nasal epithelium from a rabbit of group 4 (septicemia). (a) Severe mitochondrial vacuolation (arrows) of several ciliated cells. 10000x. (b) Severe vacuolation of the apical cytoplasm of a ciliated cell. Some vacuole (v) contain myelin-like material. 11500x.

Animals in this group had the most intense and numerous lesions. In two of the rabbits, copious amounts of detritus, inflammatory cells, and mucus accumulated in the lumen, but this accumulation was moderate in the remaining rabbit. A small number of bacteria were also observed free in the lumen. The intercellular spaces were severely dilated (Figure 3), with some epithelial cells detaching multifocally. The cytoplasm of the epithelial cells was severely vacuolated. Occasionally, these changes were associated with migrating inflammatory cells, most of which were heterophils. The number of heterophils was moderate in two cases, severe in the other animal. The number of goblet cells did not appear to increase in two animals, but they were found to be moderately activated and discharging their contents into the lumen. In the remaining animal, the number of these cells was drastically increased. Ultrastructurally, many heterophils were found migrating through the epithelium. Some of these cells were degranulated and associated with the widening of intercellular spaces, on occasion, completely separating the epithelial cells (Figure 4). Focally, the epithelial cells were severely desquamated and vacuolated. Three types of vacuoles were observed: one corresponded to swollen or burst mitochondria (Figure 5(a)), another was completely phagocytic in appearance and contained digested material, and the third contained material resembling “myelin figures” (Figure 5(b)). Isolated dead cells, including epithelial and inflammatory cells, were also observed to varying degrees, accumulating with large amounts of mucus, cellular detritus, and inflammatory cells in the lumen. Few double-walled bacteria were found, as they did not adhere to any epithelial structure.

#### 3.2.4. Group 4, Rhinitic Animals (*n* = 3)

The lesions differed in quantity but not in quality from those of septicemic rabbits, though some of them were milder in the rhinitic rabbits. Migrating inflammatory cells were associated with widened interepithelial spaces and cytoplasmic vacuolation, with both changes ranging from mild to moderate. More significant in the epithelium was the increased number of goblet cells, which in some cases were the only cells in a large area. Two rabbits were severely affected, while the remaining one was moderately so. Ultrastructurally, the goblet cells displayed high activity. They were larger than and out-numbered the ciliated cells over large areas of tissue. In between, lymphocytes and heterophils migrated through the intercellular spaces. 

## 4. Discussion

*P. multocida* is the main pathogen associated with rhinitis, pneumonia, and septicemia in rabbits. The primary ultrastructural changes in some regions of the upper respiratory tract of rabbits have been described in experimentally infected animals [13], and a detailed description of the lesions caused by natural infection in the lung has been documented [17]. We did not, however, find a characterization of the normal histology and ultrastructure of the nasal septum of rabbits during the first weeks of life or of the lesions in the same region during the natural course of two forms of the disease as reported in this study. In addition, a thorough understanding of the pathogenesis of this disease is still lacking. Aside from the normal histology and ultrastructure of the nasal septum, we discuss here the meaning of the ultrastructural lesions during the course of disease in this region. Two such findings, in our opinion, are of special relevance for the pathogenesis of disease in the nasal cavity: the interepithelial spaces and the activity of goblet cells.

The presence of interepithelial spaces in both healthy and diseased rabbits was one of the most important findings of group three. In healthy animals, however, such spaces were always restricted to the basal region of the epithelium (Figures 1(a) and 1(b)) and were never observed separating the cells at their apical junctions, as occurred in diseased rabbits. In addition to the dilatations being more severe in the latter, they were frequently complete, spanning the whole extent of the epithelium such that direct exposure of the basal lamina to the lumen of the nasal cavity was commonly observed (Figure 3). These spaces had been previously documented in healthy and diseased rabbits in the same region by histological techniques [14]. Ultrastructurally, they have also been reported in the upper airways in humans (nasal cavity and trachea), hamster (nasal cavity), rats (nasal cavity and trachea), and rabbit (trachea), but their function is unknown [18–21]. Grevers [22] suggests that it represents a physiological state, whereby fluids from the fenestrated vessels of the propria leak through the epithelium to the lumen, or that they are the natural conduits for inflammatory cells, both of which would transiently dilate the spaces. Recently, a possible explanation for the cause and mechanism of formation of these spaces under inflammatory conditions and their pathophysiological meaning was proposed by McClenahan et al. [23]. Experimentally, they showed that monolayers of bovine pulmonary epithelial cells (BPEs) underwent striking morphological changes when exposed to ATP and that these changes consisted almost exclusively of separation of the cells and an increase in permeability. During *Mannheimia haemolytica* infection in cows, one of the hallmark pathological changes is the extensive leakage of vascular products into the lung interstitium and air spaces [24–26]. Lipopolysaccharide (LPS) can induce similar permeability changes in endothelial cells in vitro, but the effects require more than 12 h [27]. Because leakage of vascular products into the lung occurs earlier in the course of *M. haemolytica* infection, McClenahan et al. [23] postulated the need for a molecule acting in the early phases of the disease, and one such molecule is ATP. ATP is known to induce anion secretion in airway epithelial cells and surfactant secretion in alveolar epithelial cells, both of which help to remove noxious particles from the lung [28–30]. Different sources of ATP during inflammation have been detected, including bacteria, apoptotic and necrotic host cells, activated epithelial cells, and macrophages [31–34]. 

At least two potential mechanisms would explain the changes in the permeability and morphology of the cell monolayers: changes in the actin cytoskeleton or modifications to the tight junction proteins. It is likely that actin-myosin contraction creates intercellular gaps in both epithelial and endothelial cell monolayers [23, 30, 35], and the modification of tight junction proteins has been best studied during leukocyte migration and release of proteases through endothelial monolayers [36], but tight and adherens junction protein modifications also occur in the absence of protease release [37]. All of these results could explain our own findings. Although activated leukocytes were found migrating through the epithelial cells of the nasal septum, and on occasion they correlated with dying cells, it was also common to find gaps between the apical junctions of epithelial cells in the absence of leukocytes or even without evidence of damage to the cells (Figure 3). 

For the mucociliary apparatus to function properly as the first defense mechanism against different invaders, it is important that the fluid surrounding the cilia as well as the viscoelasticity of the mucus blanket over the cilia maintains a correct hydration and concentration of electrolytes. However, both of these defense responses are altered during infectious and inflammatory reactions. Three mechanisms for water secretion onto the surface of the airway epithelium have been elucidated: by serous cells in the surface epithelium (Rogers et al., 1993 in [38]), by serous cells in submucosal glands, and by plasma transudate. Under normal circumstances, water is secreted by the first two mechanisms, while local irritation causes plasma transudation to become an important contributor to the volume of the airway surface liquid [38]. The breaches in the apical junctions observed in this work might represent a rise in the plasma transudation necessary to maintain the correct hydration and transit of larger quantities of secreted mucus, reflected in turn by the increased number and activity of goblet cells that would otherwise accumulate as in chronic conditions such as cystic fibrosis in humans [38]. In our case, we could demonstrate that both mechanisms are responding to the bacterium and potentially to some of its products, including LPS, from the earliest phases of the infection. This reaction might not be restricted only to rabbits suffering the open form of the infection but could also be important in healthy animals just prior to the most susceptible age for developing clinical signs (50 days). 

Despite that the animals of group 3 were taken during the age of most frequent development of the respiratory entity, none of them showed clinical signs of the disease; although it is not possible to state that they would not develop the disease later, there was a significant change in rabbits of group 4 that was negligible or absent in the healthy groups, namely, the number and the activity of goblet cells, making this event unlikely. Recent findings from our group [39] demonstrated that one of the first responses of the respiratory epithelium of nasal septa of rabbit fetus experimentally exposed to *P. multocida*, even as soon as two hours of exposure, was the increased number and activity of goblet cells, which makes this response one of the hallmarks of the lesions induced by these bacteria. 

Hypertrophy, hyperplasia, and metaplasia of the mucous cells in the epithelium and of the mucous and serous glands in the submucosa are associated with oversecretion of mucus and are some of the most prevalent lesions in rhinitis, sinusitis, and tracheobronchitis [40]. The intranasal instillation of endotoxin in rats increased the infiltration of heterophils and the quantity of mucosubstances in the epithelium [41]. In the diseased rabbits in our study, the number and the activity of goblet cells were increased relative to the healthy state, and many of these cells discharged their contents, resulting in mucus accumulation in the lumen of the organ. Heterophils play a central role in the degranulation of goblet cells in animals sensitized to ovalbumin [42]. This degranulation can be explained by the release of elastase from the azurophilic granules of heterophils in the regions where goblet cells are degranulating. Elastase is a powerful secretagogue on the glands and goblet cells of the airways [43]. 

The cytoplasmic vacuolation of epithelial cells in the nasal cavity was more severe in diseased animals, particularly, those with septicemia (Figure 5(a)). This phenomenon was also observed in healthy animals from group 3. This change was associated with a moderate-to-severe infiltration of heterophils into the propria and the epithelium. Such an association would suggest that the passage and degranulation of these cells in the interepithelial space, especially when activated during pathological events, would induce irreversible damage to the cell membranes, causing their degeneration (vacuolation), death, and desquamation [44]. 

In our study, three patterns of vacuolation were observed in the epithelial cells: one corresponded to swollen or exploded mitochondria, the most common one had the appearance of digested cellular components, and the final one contained material resembling myelin figures. These changes might be induced by the *P. multocida* LPS. Kang et al. [45] found aggregates of LPS in the cytoplasm, phagosomes, mitochondria, and nucleus of alveolar macrophages incubated with LPS. They proposed that the LPS caused a functional perturbation of the pilocytic vesicles, phagocytic vacuoles, cytoplasm, mitochondria, endoplasmic reticulum, Golgi apparatus, and nucleus of these cells [45]. The accumulation of lipoprotein membranes in a concentric lamellar fashion results in the formation of myelin figures, which can be the product of a degenerative process, where death or damage to the cellular organelles has taken place, and their membranes remain in residual bodies [46]. In our case, the myelin figures were found occupying the same locations as the swollen mitochondria; thus, these two types of vacuoles could both correspond to the same phenomena: damage to the mitochondria. The vacuoles containing myelin figures, however, were larger than the mitochondria, so it could also be possible that they were phagocytic vacuoles in which those figures would also be formed when the cell phagocytizes structures that contain membranes and/or products with lipoprotein composition, such as bacteria or its LPS. When monocytes ingest LPS, it forms lipidic bilayers and micelles and is observed within the phagocytic vacuoles forming lamellar micelles or myelin figures [47, 48]. This last finding would favor the hypothesis that, in our study, this type of vacuole corresponds to a phagocytic vacuole.

In addition, there appeared to be a direct relationship between the number and size of vacuoles, the extension of intercellular spaces, and the number of migrating heterophils. The heterophils of the diseased rabbits seemed to be degranulating, which was reflected by the decreased quantity and smaller size of their granules, leaving clear spaces in the cytoplasm. Klut et al. [49] documented a loss of electrodensity and size of the granules in rabbit heterophils exposed to LPS. 

In asthma, bronchial hyperreactivity, infectious pneumonia, endotoxemia, and so forth, authors have demonstrated that heterophils and eosinophils are responsible for tissue damage [44, 50–53]. Intranasal instillation of LPS induces a severe infiltration and migration of heterophils into the propria and their passage through the mucosa [40, 41, 50, 52]. LPS injected intraperitoneally in rabbits induced an increase in circulating IL-8 and the number of activated heterophils, which overexpress the CD11/CD18 integrins. A severe injury to lung endothelial and epithelial cells by migrating heterophils was also evident, and the injury was related to the degranulation of heterophils and liberation of their granule content into the extra cellular space [49, 52].

Ultrastructural changes similar to those observed here in the upper airways of rabbits inoculated experimentally with *P. multocida* A3 were obtained by Al-Haddawi et al. [11]. The primary lesions exhibited cellular and mitochondrial swelling, dilatation and degeneration of the cristae, RER distention, cytoplasm vacuolation, and uneven nuclear membranes. Goblet cell hyperplasia and hypertrophy were also found, and heterophils were associated with the degenerated epithelial cells. They hypothesize that the epithelial changes are caused by the inflammatory cells when they migrate through the epithelium, which we also support. However, in contrast to our findings, they reported *Pasteurella* adhering to degenerated cilia, the microvilli, and the mucus [11]. In several works with rabbits expressing the natural form of the disease [17, 54], and in the present study, there have been few bacteria in the diseased rabbits, and these few were only observed adhering to the mucus, never to cell structures. Differences in the models between Al-Haddawi et al.'s work [11] and ours could explain the divergences. While Al-Haddawi et al. [11] inoculated a large number of viable bacteria (10^9^ UFC) intranasally in rabbits free of *P. multocida*, our animals developed the disease naturally. 

Our findings do not guarantee that *P. multocida* was the only, or even the main, etiologic factor of the respiratory disease in the rabbits described here because there were few of these cells and they did not adhere to the epithelium. The TEM technique is highly sensitive to detect this host-microorganism interaction. Even more, cell damage was not associated with the presence of microorganisms. In our opinion, more attention should be paid to some of its toxins, such as LPS, rather than to bacterial proliferation alone. 

Endotoxins are commonly found in different environments [41, 55], and the continuous inhalation of these molecules is an important etiologic factor in occupational diseases [41, 56–58]. Due to the high population density at which the rabbits are kept, the air around them is highly charged with residues, dust, ammonia, and even bacteria and their toxins. The constant bombardment of these particles over the epithelium could be providing a physical or chemical stimulus capable of inducing an inflammatory response in the epithelial cells and the posterior proliferation of mucous cells and other lesions in the rabbit's nasal cavity [58].

In summary, to our knowledge, this is the first description of the nasal cavity of healthy rabbits and of rabbits afflicted with the spontaneous form of the disease caused by *P. multocida* by TEM. The importance of plasma transudation through the epithelial cells of the rise in mucus production by goblet cells to defend against the infection is thoroughly discussed. We also discuss the meaning of the small number of bacteria found in this work. Findings such as the migration of heterophils through the epithelium and vacuole formation are also taken into account. 

## Figures and Tables

**Table 1:** TEM findings. Severity of changes in cells of respiratory epithelium of nasal cavity.

Changes/groups	Intercellular spaces	Vacuoles in cytoplasm	Detritus	Heterophils	Bacteria	Activity goblet cells	No. of goblet cells
Group 1	+	++	−	+	−	−	+
Group 2	+	−	−	+	−	−	+
Group 3	++ +++	++ +++	−	++		−	+
Group 4 S	+++	+++	++^1^ +++^2^	++^2^ +++^1^	+	++^2^	+++^1^
Group 4 R	+ ++	+ ++		++		+++	++^1^ +++^2^

− No change; + light; ++ moderate; +++ severe; ^1^number of animals; S: Septicemic; R: Rhinitic.
